# Genetic and pharmacological correction of aberrant dopamine synthesis using patient iPSCs with BH4 metabolism disorders 

**DOI:** 10.1093/hmg/ddw339

**Published:** 2016-10-18

**Authors:** Taizo Ishikawa, Keiko Imamura, Takayuki Kondo, Yasushi Koshiba, Satoshi Hara, Hiroshi Ichinose, Mahoko Furujo, Masako Kinoshita, Tomoko Oeda, Jun Takahashi, Ryosuke Takahashi, Haruhisa Inoue

**Affiliations:** 1 Center for iPS Cell Research and Application (CiRA), Kyoto University, 53 Kawahara-cho, Shogoin, Sakyo-ku, Kyoto, Japan; 2 Sumitomo Dainippon Pharma, 3-1-98 Kasugadenaka, Konohana-ku, Osaka, Japan; 3 Department of Neurology, Kyoto University Graduate School of Medicine, 54 Kawahara-cho, Shogoin, Sakyo-ku, Kyoto, Japan; 4 School of Life Science and Technology, Tokyo Institute of Technology, Yokohama, Japan; 5 Department of Pediatrics, Okayama Medical Center, National Hospital Organization, Okayama, Japan; 6 Department of Neurology, Utano National Hospital, National Hospital Organization, Kyoto, Japan

## Abstract

Dopamine (DA) is a neurotransmitter in the brain, playing a central role in several disease conditions, including tetrahydrobiopterin (BH4) metabolism disorders and Parkinson’s disease (PD). BH4 metabolism disorders present a variety of clinical manifestations including motor disturbance via altered DA metabolism, since BH4 is a cofactor for tyrosine hydroxylase (TH), a rate-limiting enzyme for DA synthesis. Genetically, BH4 metabolism disorders are, in an autosomal recessive pattern, caused by a variant in genes encoding enzymes for BH4 synthesis or recycling, including 6-pyruvoyltetrahydropterin synthase (PTPS) or dihydropteridine reductase (DHPR), respectively. Although BH4 metabolism disorders and its metabolisms have been studied, it is unclear how gene variants cause aberrant DA synthesis in patient neurons. Here, we generated induced pluripotent stem cells (iPSCs) from BH4 metabolism disorder patients with PTPS or DHPR variants, corrected the gene variant in the iPSCs using the CRISPR/Cas9 system, and differentiated the BH4 metabolism disorder patient- and isogenic control iPSCs into midbrain DA neurons. We found that by the gene correction, the BH4 amount, TH protein level and extracellular DA level were restored in DA neuronal culture using PTPS deficiency iPSCs. Furthermore, the pharmacological correction by BH4 precursor sepiapterin treatment also improved the phenotypes of PTPS deficiency. These results suggest that patient iPSCs with BH4 metabolism disorders provide an opportunity for screening substances for treating aberrant DA synthesis-related disorders.

## Introduction

Dopamine (DA), a physiological chemical of the catecholamine family, is involved in several important aspects of brain function, including motor control and reward. DA deficiency causes various disease conditions including one of the major neurodegenerative diseases, Parkinson’s disease (PD). A rate-limiting enzyme for DA synthesis is tyrosine hydroxylase (TH). Lack of tetrahydrobiopterin (BH4; a cofactor of TH) dysregulates DA synthesis and causes movement disorders and psychiatric disorders (1).

BH4 is synthesized from guanosine triphosphate (GTP) by a set of reactions involving three enzymes, GTP cyclohydrolase I (GTPCH), which is the first and rate-limiting enzyme, 6-pyruvoyltetrahydropterin synthase (PTPS), and sepiapterin reductase (SR), and is recycled by two enzymes, pterin-4a-carbinolamine dehydratase (PCD) and dihydropteridine reductase (DHPR) (2). Variants in the genes encoding these enzymes cause BH4 metabolism disorders (PTPS deficiency; OMIM entry #261640, DHPR deficiency; OMIM entry #261630) (1). Since BH4 is also a cofactor of phenylalanine hydroxylase (PAH), BH4 metabolism disorders are associated with hyperphenylalaninemia (HPA). Several BH4 metabolism disorder model mice including PTPS knock-out mice have been created, and they developed aberrant DA synthesis (3,4). BH4 administration in these model mice partially restored reduced DA levels in the brain (3,4). In human, the therapeutic efficacy of BH4 itself on neurological symptoms has been limited, although patients with BH4 metabolism disorders are also treated with BH4 supplementation for HPA caused by ineffective PAH in liver (1). Therefore, it is still unclear how the gene variant causes aberrant DA synthesis in patient neurons, and what substance could be used to attenuate its dysregulation.

Recent advances in human induced pluripotent stem cell (iPSC) technology have provided access to unlimited numbers of patient neurons (5). This technology has been used to generate patient neuronal models of PD and recapitulate key features of the disease (6,7). Furthermore, the generation of genetically matched isogenic iPSCs using gene-editing techniques provides ideal control, since it eliminates differences in genetic background and provides a clear conclusion regarding gene variant-specific phenotypes (8,9).

Here, we generated BH4 metabolism disorder patient-derived iPSCs with gene variants in PTPS or DHPR, which are involved in BH4 synthesis or regeneration, and carried out gene correction of the variant in the iPSCs by one of the gene editing techniques, the CRISPR/Cas9 system, to obtain genetically matched non-diseased controls. We differentiated iPSCs into DA neurons and investigated the DA synthesis dysregulation, treated by gene correction or BH4 supplementation. Furthermore, we tested sepiapterin, which is a substrate of the salvage pathway in BH4 synthesis, as a promising substance.

## Results

### Generation of BH4 metabolism disorder-derived iPSCs and gene correction of variant

**Figure 1:**
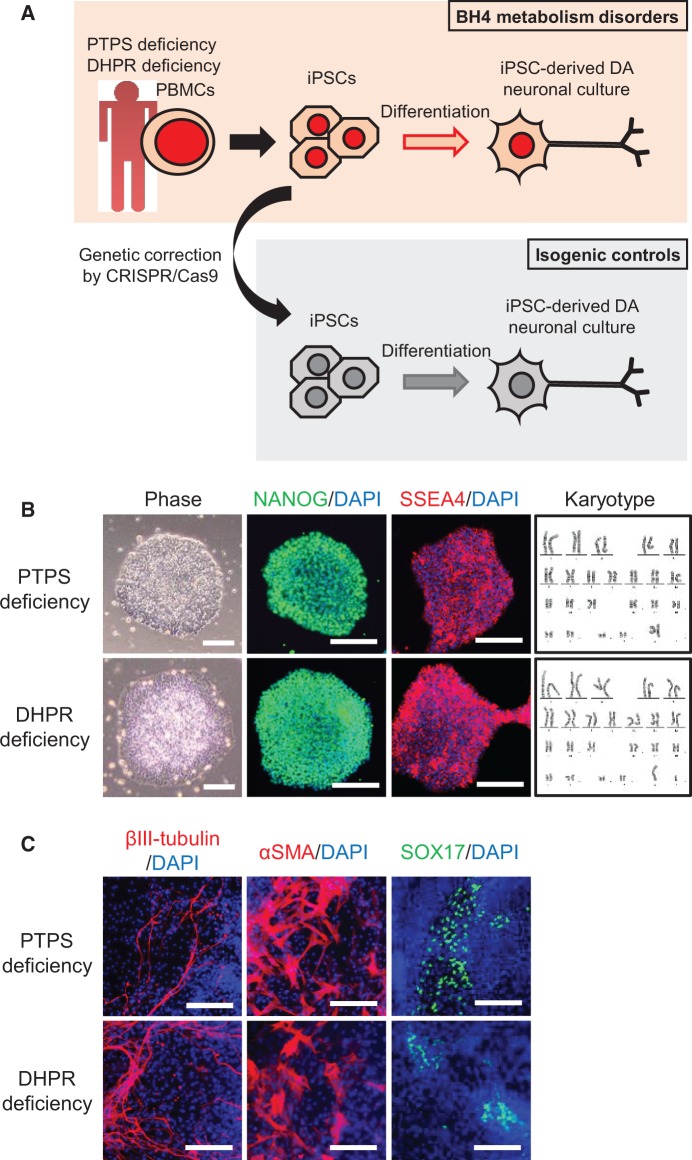
Generation and characterization of BH4 metabolism disorder iPSCs. **(A)** Schematic of strategy for comparing DA neuronal culture using iPSCs from BH4 metabolism disorder patients with that using genetically corrected iPSCs. **(B)** Phase-contrast images, immunocytochemistry for pluripotency markers NANOG and SSEA4, and karyotype analysis in PTPS deficiency iPSCs and DHPR deficiency iPSCs. Scale bars: 100 μm. **(C)***In vitro* differentiation of PTPS deficiency iPSCs and DHPR deficiency iPSCs to three germ layers: βIII-tubulin (ectoderm), αSMA (mesoderm), and SOX17 (endoderm). Scale bars: 100 μm.

BH4 metabolism disorder patients present psychomotor retardation and abnormal movements associated with a DA deficiency. The generation of patient-derived iPSCs and gene correction of the variant together provide an opportunity to obtain mechanistic insights and screening for new therapeutic compounds using iPSC-derived disease-affected cells (10,11). To investigate gene variant-specific disease phenotypes in DA neuronal culture using iPSCs from patients with BH4 metabolism disorders (Fig. 1A), we initially generated iPSCs from peripheral blood mononuclear cells of PTPS- or DHPR deficiency patients. Both iPSCs were positively stained for pluripotency markers, displayed a normal karyotype (Fig. 1B), and were able to differentiate into cells of three germ layers *in vitro* (Fig. 1C).

**Figure 2:**
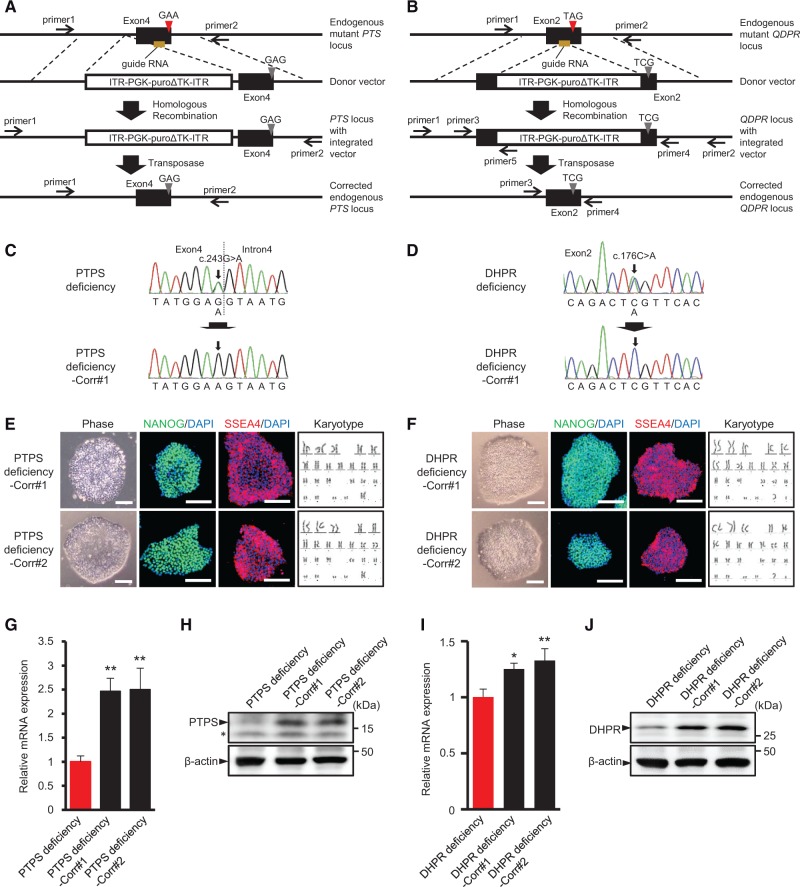
Gene correction of variant in BH4 metabolism disorder iPSCs. **(A,B)** Schematic of strategy used for CRISPR/Cas9-mediated gene correction of *PTS* c.243G>A variant (A) and *QDPR* c.176C>A variant (B). Red triangle denotes the gene variant. Grey triangle denotes the corrected gene variant. **(C,D)** Sequencing of genomic *PTS* locus in indicated PTPS iPSCs (C) and genomic *QDPR* locus in indicated DHPR iPSCs (D). The corrected gene variant is marked with an arrow. **(E,F)** Phase-contrast images, immunocytochemistry for pluripotency markers NANOG and SSEA4, and karyotype analysis in PTPS deficiency-corrected iPSCs (E) and DHPR deficiency-corrected iPSCs (F). Scale bars: 100 μm. **(G,I)** Gene expression analysis of PTPS deficiency iPSCs and PTPS deficiency-corrected iPSCs for PTPS mRNA (G) and DHPR deficiency iPSCs and DHPR deficiency-corrected iPSCs for DHPR mRNA (I). *GAPDH* was used as normalizer. Data represent mean ± S.D. of *n = * 3. *P <  0.05, **P <  0.01, one-way ANOVA with Dunnett’s tests compared to patient iPSCs. **(H,J)** Western blot analysis of PTPS deficiency iPSCs and PTPS deficiency-corrected iPSCs for PTPS and β-actin protein levels (H) and DHPR deficiency iPSCs and DHPR deficiency-corrected iPSCs for DHPR and β-actin protein levels (J). Asterisk indicates non-specific band.

Recent progress in human gene editing using the CRISPR/Cas9 system allows for the gene correction of a disease-causing variant in iPSCs, and thereby the generation of genetically matched control cell lines (12,13). Therefore, to correct the causative gene variant in BH4 metabolism disorder iPSCs, we used the CRISPR/Cas9 and *piggyBac* transposase system. The guide RNA (gRNA) was designed to target sequences in the vicinity of the c.243G > A variant in the endogenous *PTS* gene in PTPS deficiency (Fig. 2A) or the c.176C > A variant in the endogenous *QDPR* gene in DHPR deficiency (Fig. 2B). Donor vector contained a PGK promoter-driven Puro-ΔTK cassette flanked by *piggyBac* inverted terminal repeats (ITR) along with the homology arms with the corrected *PTS* or *QDPR* sequence for integration into the endogenous genomic locus (Fig. 2A and B). The BH4 metabolism disorder iPSCs were co-electroporated with the gRNA vector, donor vector, and Cas9 vector, followed by puromycin selection. Puromycin-resistant colonies were selected and confirmed for cassette integration by PCR amplification (Supplementary Material, Fig. S1A and C). This selection cassette was excised using the *piggyBac* transposase system, which allows seamless removal of the *piggyBac* repeats-flanked sequence. Integration-positive clones were transiently electroporated by a transposase expression vector, followed by negative selection with 1-(2-deoxy-2-fluoro-1-D-arabinofuranosyl)-5-iodouracil (FIAU). FIAU-resistant colonies were screened by PCR amplification (Supplementary Material, Fig. S1B and D). Correction of the gene variant and seamless *piggyBac* excision in the corrected iPSCs (PTPS deficiency-Corr and DHPR deficiency-Corr) were confirmed by sequence analyses (Fig. 2C and D). Corrected iPSCs displayed embryonic stem (ES) cell-like morphology and expressed the pluripotency markers (Fig. 2E and F). Chromosomal analysis revealed a normal karyotype (Fig. 2E and F). All corrected iPSCs also could differentiate into all three germ layers *in vitro* (Supplementary Material, Fig. S2A). PTPS mRNA and protein level were greatly increased in PTPS deficiency-corrected iPSCs compared to PTPS deficiency iPSCs (Fig. 2G and H). Similarly, DHPR mRNA and protein level were increased in DHPR deficiency-corrected iPSCs compared to DHPR deficiency iPSCs (Fig. 2I and J). The c.243G > A variant in *PTS* gene reduces the PTPS protein level via reduction of full-length PTPS mRNA by splicing error (14). On the other hand, the c.176C > A variant in *QDPR* gene reduces the DHPR protein level via creation of a premature stop codon by nonsense variant. Enzyme activity assay showed that PTPS activity was increased in PTPS deficiency-corrected iPSCs compared to PTPS deficiency iPSCs (Supplementary Material, Fig. S2C). On the other hand, DHPR activity in DHPR deficiency iPSCs and DHPR deficiency-corrected iPSCs was undetectable in colorimetric assay.

### BH4 metabolism disorder iPSC-derived DA neuronal cultures show decreased TH protein level and extracellular DA level

**Figure 3:**
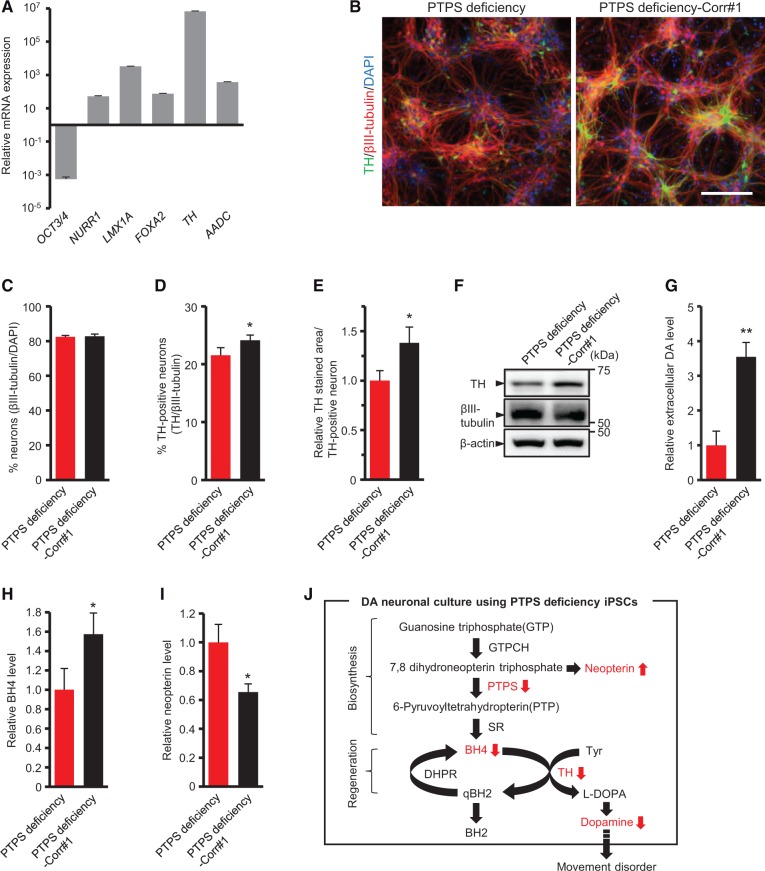
PTPS deficiency iPSC-derived DA neuronal culture shows decreased TH protein level and extracellular DA level. **(A)** Gene expression analysis for key midbrain DA neuron markers. *GAPDH* was used as normalizer. The expression level of undifferentiated cells (day 0) was set at 1. **(B)** DA neuronal cultures using PTPS deficiency iPSCs and PTPS deficiency-corrected iPSCs were stained for TH, the marker for neuron βIII-tubulin and DAPI. Scale bar: 100 μm. **(C–E)** Percentage of βIII-tubulin-positive neurons in total cells (C), percentage of TH-positive neurons in βIII-tubulin-positive neurons (D) and the relative area of TH-positive neurons (E) in DA neuronal cultures using PTPS deficiency iPSCs and PTPS deficiency-corrected iPSCs. **(F)** Western blot analysis of DA neuronal cultures using PTPS deficiency iPSCs and PTPS deficiency-corrected iPSCs for TH, βIII-tubulin and β-actin protein levels. **(G)** Relative level of high potassium evoked-extracellular DA release in DA neuronal cultures using PTPS deficiency iPSCs and PTPS deficiency-corrected iPSCs. **(H,I)** Pterin metabolic profiles of DA neuronal cultures using PTPS deficiency iPSCs and PTPS deficiency-corrected iPSCs: BH4 (H), Neopterin (I). **(J)** Metabolic pathway of BH4 and DA synthesis in PTPS deficiency. c.243G > A variant in *PTS* gene causes reductions of BH4 amount, TH protein level, and extracellular DA level in DA neuronal culture. Enzymes are indicated by the following abbreviations: GTPCH, GTP cyclohydrolase I; PTPS, 6-pyruvoyltetrahydropterin synthase; SR, sepiapterin reductase; DHPR, dihydropteridine reductase; TH, tyrosine hydroxylase. Quantification represents mean ± S.D. of n = 3. *P < 0.05, **P < 0.01, Student’s *t* test.

To investigate the phenotypes of disease-affected cells, we differentiated iPSCs into DA neurons. Differentiation to DA neurons was carried out by using dual SMAD inhibition and the floor plate induction protocol as previously reported (15,16). At differentiation day 42, this protocol induced strong expression of *NURR1*, *FOXA2*, *LMX1A*, *TH*, *AADC*, indicating establishment of midbrain DA neurons (Fig. 3A). Meanwhile, *OCT4*, one of the genes that regulate the pluripotency of stem cells, was down-regulated (Fig. 3A). Further, a high-performance liquid chromatography (HPLC) analysis revealed that the differentiated DA neuronal culture was functional in extracellular DA release (Supplementary Material, Fig. S2D).

PTPS deficiency is the most frequent form (60%) of BH4 metabolism disorders (1). Based on the above protocol, we differentiated PTPS deficiency iPSCs and PTPS deficiency-corrected iPSCs into DA neurons. Although the percentage of βIII-tubulin-positive neurons in total cells was not significantly different among DA neuronal cultures, the percentage of TH-positive neurons among βIII-tubulin-positive neurons was slightly reduced in DA neuronal culture using PTPS deficiency iPSCs compared to that using PTPS deficiency-corrected iPSCs (Fig. 3B–D). In addition, the area of TH-positive neurons and the TH protein level were greatly reduced in DA neuronal culture using PTPS deficiency iPSCs compared to that using PTPS deficiency-corrected iPSCs (Fig. 3B,E and F), and the extracellular DA level was also reduced in DA neuronal culture using PTPS deficiency iPSCs (Fig. 3G). We also determined the metabolic profile of pterins including BH4 and neopterin by HPLC in iPSC-derived DA neuronal culture. In DA neuronal culture using PTPS deficiency iPSCs, the BH4 amount was reduced (Fig. 3H) and the neopterin level was increased (Fig. 3I). These results showed that c.243G > A variant in *PTS* gene causes reductions in the BH4 amount, TH protein level and extracellular DA level in DA neuronal culture (Fig. 3J). The observed abnormal phenotypes in DA neuronal culture using PTPS deficiency iPSCs are consistent with the symptoms of motor disturbance and reduced homovanillic acid (HVA), one of the catecholamine metabolites in cerebrospinal fluid (CSF), which are found in patients with PTPS deficiency.

Next, we differentiated DHPR deficiency iPSCs and DHPR deficiency-corrected iPSCs into DA neurons in the same way. The TH protein level was slightly reduced in DA neuronal culture using DHPR deficiency iPSCs (Supplementary Material, Fig. S3A), and the extracellular DA level was significantly reduced in DA neuronal culture using DHPR deficiency iPSCs (Supplementary Material, Fig. S3B). Pterin analysis showed that, unlike the results of DA neuronal cultures using PTPS deficiency iPSCs and PTPS deficiency-corrected iPSCs, there were no differences in the amounts of BH4 and neopterin between DA neuronal cultures using DHPR deficiency iPSCs and DHPR deficiency-corrected iPSCs (Supplementary Material, Fig. S3C and D). Further analysis showed a large increase of total biopterin caused by increases of dihydrobiopterin (BH2) and biopterin in DA neuronal culture using DHPR deficiency iPSCs (Supplementary Material, Fig. S3E). The observed reduced DA level and increased total biopterin in DA neuronal culture using DHPR deficiency iPSCs are also consistent with profiles of HVA and pterins in patients’ CSF with DHPR deficiency (17).

### Sepiapterin improves the TH protein level and extracellular DA level in PTPS deficiency iPSC-derived DA neuronal culture

**Figure 4:**
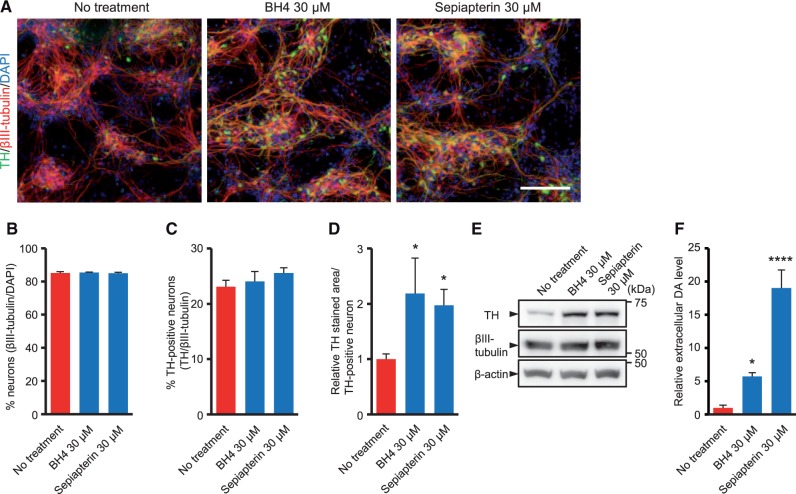
Sepiapterin improves TH protein level and extracellular DA level in PTPS deficiency iPSC-derived DA neuronal culture. **(A)** PTPS deficiency iPSC-derived DA neuronal cultures treated with DMSO, 30 μM BH4 or 30 μM sepiapterin were stained for TH, the marker for neuron βIII-tubulin and DAPI. Scale bar: 100 μm. **(B–D)** Percentage of βIII-tubulin-positive neurons in total cells (B), percentage of TH-positive neurons in βIII-tubulin-positive neurons (C), and relative area of TH-positive neurons (D) in PTPS deficiency iPSC-derived DA neuronal culture treated with DMSO, 30 μM BH4 or 30 μM sepiapterin. **(E)** Western blot analysis of PTPS deficiency iPSC-derived DA neuronal culture treated with DMSO, 30 μM BH4 or 30 μM sepiapterin for TH, βIII-tubulin and β-actin protein levels. **(F)** Relative level of high potassium evoked-extracellular DA release in PTPS deficiency iPSC-derived DA neuronal culture treated with DMSO, 30 μM BH4 or 30 μM sepiapterin. Quantification represents mean ± S.D. of *n = * 3. *P <  0.05, ****P <  0.0001, one-way ANOVA with Dunnett’s tests compared to non-treatment control.

Administration of BH4 is beneficial in PTPS knock-out mice with aberrant DA synthesis (3). To evaluate whether BH4 treatment can attenuate the disease phenotypes in human DA neuronal culture with PTPS deficiency, we treated it with BH4 for five days following the differentiation period. We also tested sepiapterin, a substrate of the salvage pathway of BH4 synthesis, in the same manner. Although the chemical structures of both substrates are very similar, sepiapterin can more effectively raise the tissue BH4 amount than BH4 itself (18). Immunocytochemistry revealed that the intensity of the TH staining signal in PTPS deficiency iPSC-derived DA neuronal culture treated with BH4 or sepiapterin was significantly increased compared to non-treatment control (Fig. 4A). Although the percentages of βIII-tubulin-positive neurons and TH-positive neurons were not significantly different regardless of the treatment, increased TH-stained area and TH protein level with significant neurite localization of TH was observed in BH4- or sepiapterin-treated DA neuronal culture (Fig. 4A–E). In addition, the extracellular DA levels in PTPS deficiency iPSC-derived DA neuronal culture treated with BH4 or sepiapterin were significantly higher than that of non-treatment control (Fig. 4F). We found that sepiapterin increased the extracellular DA level to a greater extent than BH4 at the same concentration (Fig. 4F).

Traditional treatment of DA-related neurological symptoms in BH4 metabolism disorders is L-DOPA therapy. Sepiapterin showed an additive effect with L-DOPA in extracellular DA release assay, rather than in the TH protein level (Supplementary Material, Fig. S4A and B). Furthermore, we found that sepiapterin treatment also increased the intensity of the TH staining signal and the extracellular DA level in PD iPSC-derived DA neuronal culture (Supplementary Material, Fig. S4C and D).

## Discussion

In the present study, we investigated the patient- and gene variant-specific DA synthesis dysregulation treated by gene correction or BH4 supplementation, and evaluated the efficacy of sepiapterin in human DA neuronal culture with PTPS deficiency. We initially generated iPSCs from BH4 metabolism disorder patients with PTPS or DHPR variants. The c.243G > A variant in *PTS* gene and the c.176C > A variant in *QDPR* gene were corrected using the CRISPR/Cas9 and *piggyBac* transposase system. PTPS full-length mRNA and protein level were greatly decreased in PTPS deficiency iPSCs compared to PTPS deficiency corrected iPSCs. The c.243G > A variant in *PTS* gene affects the splice donor site of exon4 and causes exon4 skipping, leading to a decreased level of full length mRNA (14). We also confirmed a splicing variant of PTPS only in patient iPSCs. On PCR amplification of PTPS cDNA, PTPS deficiency iPSCs exhibited a shorter band than the expected wild-type band (452bp) (Supplementary Material, Fig. S2B). This result suggests that the reduction of PTPS full-length mRNA caused by splicing error leads to a reduction of the PTPS protein level in PTPS deficiency iPSCs with c.243G > A variant. On the other hand, the c.176C > A variant in *QDPR* gene is a nonsense variant, resulting in a reduced DHPR protein level. The nonsense-mediated mRNA decay may cause a decreased level of DHPR mRNA in DHPR deficiency iPSCs. We also showed that PTPS activity in PTPS deficiency iPSCs was lower than that in PTPS deficiency-corrected iPSCs. Although DHPR activity in DHPR deficiency iPSCs and DHPR deficiency-corrected iPSCs was undetectable, an increase of oxidized biopterins in DA neuronal culture using DHPR deficiency iPSCs suggests that DHPR activity in DHPR deficiency cells was lower than that in DHPR deficiency-corrected cells, because low DHPR activity causes inefficient reduction of *quinonoid* dihydrobiopterin (qBH2) to BH4, resulting in increases of BH2 and biopterin.

DA neuronal culture using PTPS deficiency iPSCs showed decreases in TH protein level, extracellular DA level and BH4 amount compared to that using PTPS deficiency-corrected iPSCs. Reduced CSF biopterin and HVA in patients with PTPS deficiency, have been reported (1). A reduction in the TH protein level in the striatum has also been reported in autopsy studies of patients with Dopa-responsive dystonia (DRD), which is caused by an autosomal-dominant defect in the *GCH1* gene encoding the rate-limiting enzyme for BH4 synthesis (19,20). Therefore, this result shows that the human PTPS deficiency iPSC-derived disease model recapitulates the disease phenotypes in the patient brain with impairment of BH4 synthesis including DRD, and the c.243G > A variant in *PTS* gene is enough to cause them. One possible mechanism for the TH protein level reduction is that BH4 or DA deficiency induces phosphorylation of TH, which in turn leads to its degradation through an ubiquitin-proteasome pathway (21). On the other hand, DA neuronal culture using DHPR deficiency iPSCs did not show a decrease in BH4 amount compared to that using DHPR deficiency-corrected iPSCs. This result indicates that BH4 was regenerated via an alternative pathway and/or that the biosynthesis of BH4 was increased in DA neuronal culture using DHPR deficiency iPSCs. Rather, the total biopterin amounts including BH2 were increased. A normal BH4 amount and an elevated BH2 amount in CSF of DHPR deficiency patients have been reported, consistent with our observation in DA neuronal culture using DHPR deficiency iPSCs. Although the BH4 amount did not differ between DA neuronal cultures using DHPR deficiency iPSCs and DHPR deficiency-corrected iPSCs, since it was reported that BH2 can act as an inhibitor of the aromatic amino acid hydroxylase (22), accumulated BH2 in DA neuronal culture using DHPR deficiency iPSCs may lead reduced the DA level via inhibition of TH (Supplementary Material, Fig. S3F).

Current treatment of BH4 metabolism disorders is based on supplemental therapy with BH4. However, the amount of BH4 that enters the brain is still not sufficient to sustain appropriate synthesis of DA in patients, and thus concomitant therapy with neurotransmitter precursors, i.e., L-DOPA, is necessary to recover an appropriate DA level in the patient brain (1). In fact, the effect of BH4 itself on a patient DA neuronal culture remains unclear. Our results showed that aberrant DA synthesis in DA neuronal culture using iPSCs from a patient with PTPS deficiency was attenuated by BH4 supplementation after the differentiation period. Moreover, sepiapterin, a substrate of the salvage pathway of BH4 synthesis, also improved these disease phenotypes. Compared to BH4, sepiapterin is a more stable form and can efficiently pass through the plasma membrane via equilibrative nucleoside transporter 2 (ENT2) (23). We actually observed that BH4 disappeared rapidly in culture medium (Supplementary Material, Fig. S3G) and that sepiapterin increased the extracellular DA level more than BH4, with both at the same concentration. Since ENT2 is also localized in the blood-brain barrier (24), sepiapterin may be effective against aberrant DA synthesis in patient brain. However, since BH4 also has a DA releasing activity that is not dependent on the biosynthesis of DA (25), there is a possibility that intracellular DA storage has been partially lost before the DA release assay is carried out. In addition, it has been reported that dihydrofolate reductase (DHFR) activity, which is required for conversion from sepiapterin to BH4, is low in the brain (26). On the other hand, it was also reported that a patient with DHFR deficiency developed cerebral BH4 deficiency (27). Therefore, the in vivo efficacy of sepiapterin in the human brain is still unknown. Clinical studies to evaluate the efficacy of sepiapterin may be necessary.

As described above, L-DOPA is typically used to increase the DA level in the treatment of neurological symptoms of BH4 metabolism disorders. The clinical outcome of BH4 metabolism disorders is frequently worsened by the reduced efficacy of long-term L-DOPA therapy, such as diurnal symptom fluctuations (28–30). For this reason, we investigated the possibility that sepiapterin is beneficial as an adjunctive therapy with L-DOPA. Sepiapterin showed an additive effect with L-DOPA in an extracellular DA release assay, rather than in the TH protein level, due to the fact that L-DOPA is also converted to DA in TH-negative/aromatic l-amino acid decarboxylase (AADC) -positive cells.

It has been reported that familial PD iPSC-derived DA neurons with glucocerebrosidase (GBA) or Leucine-rich repeat kinase 2 (LRRK2) variant showed a reduced DA level (6,31). Although it is very limited data, we found that sepiapterin treatment also increased the intensity of the TH staining signal and the extracellular DA level in PD iPSC-derived DA neuronal culture. Since a single nucleotide polymorphism (SNP) at the *GCH1* gene encoding the rate-limiting enzyme for BH4 synthesis was associated with PD in a large-scale genome-wide association study (GWAS) meta-analysis (32), sepiapterin treatment might be more effective in PD with the SNP of *GCH1* than without the SNP of *GCH1*.

In conclusion, gene correction revealed the effect of gene variant on BH4 metabolism disorder phenotypes in DA neuronal culture, and we found that sepiapterin was useful for the treatment of BH4 metabolism disorders with impairment of BH4 synthesis in an in vitro model. The generated patient iPSCs with BH4 metabolism disorders would provide an opportunity for screening a substance such as sepiapterin to treat aberrant DA synthesis-related disorders.

## Materials and Methods

### Generation of iPSCs and cell culture

Peripheral blood mononuclear cells from a PTPS deficiency patient with compound heterozygous variant (*PTS*-c.243G > A/c.259C > T) and a DHPR deficiency patient with compound heterozygous variant (*QDPR*-c.52G > T/c.176C > A) were reprogrammed by inducing the episomal vectors carrying OCT3/4, SOX2, KLF4, L-MYC, LIN28 and p53 carboxy-terminal dominant-negative fragment (33) (Supplementary Material, Table S1). Generated iPSC clones were cultured on iMatrix-511 (Nippi, Tokyo, Japan)-coated plates with StemFit medium (Ajinomoto, Tokyo, Japan) supplemented with penicillin/streptomycin.

### Gene correction of iPSCs

iPSCs were dissociated using Tryple (Thermo Fisher Scientific, Waltham, MA), centrifuged, and resuspended in Opti-MEM containing 10 μM ROCK inhibitor, Y27632. iPSCs (1x10^6^ cells) were electroporated with Cas9, gRNA expression vector, donor vector (NEPA21, Nepagene, Ichikawa, Japan) and subsequently plated on iMatrix-511-coated plates with StemFit medium containing 10 μM Y27632. After puromycin treatment, colonies were mechanically selected. To screen genomic DNA samples for integration of the corrective sequences, the target locus was amplified by PCR. Correctly targeted iPSCs (1x10^6^ cells) were electroporated with *piggyBac* transposase expression vector, followed by selection with FIAU. Individual colonies were mechanically selected. Deletion of the *piggyBac* transposon was analysed by PCR and sequencing.

### Karyotyping and genotyping

Karyotyping was performed by the LSI Medience Corporation (Tokyo, Japan). Genotyping of variants in *PTS* and *QDPR* genes was performed by PCR amplification of genomic DNA and direct sequencing (3500xL Genetic Analyzer, Thermo Fisher Scientific).

### Dopaminergic neuronal differentiation

A feeder-free culture system was used to establish iPSCs. Dopaminergic neurons were differentiated by quick embryoid body–like aggregate method and dual SMAD inhibition by modifying previous procedures (15). Briefly, a floating culture of cell aggregations was introduced, and LDN193189, A83-01, purmorphamine, CHIR99021, fibroblast growth factor 8, brain-derived neurotrophic factor, glial cell-derived neurotrophic factor, dibutyryl cyclic AMP and ascorbic acid were added. Dopaminergic neurons were evaluated 6-7 weeks later in vitro.

### RNA extraction and quantitative-PCR

Total RNA was extracted using the RNeasy Mini kit (Qiagen, Hilden, Germany) or SuperPrep Cell Lysis Kit (Toyobo, Osaka, Japan) according to the manufacturer’s instructions. Samples were analysed for RNA content using a Nanodrop spectrophotometer (Thermo Fisher Scientific). cDNA was synthesized from 1 μg of RNA using the ReverTra Ace (Toyobo). Quantitative PCRs were carried out with SYBR Premix Ex Taq (Takara, Kusatsu, Japan) and the StepOnePlus Real Time PCR System (Thermo Fisher Scientific). The data were assessed using the 2^-ΔΔCt^ method and normalized by GAPDH expression. Primer sequences are described in Supplementary Material, Table S2. 

### Amplification of PTPS cDNA

The PTPS coding region was amplified with forward primer (5’-ATGAGCACGGAAGGTGGTG-3’) and reverse primer (5’-GCTAACCCCAATAGCTATTCTCC-3’) from PTPS deficiency iPSC cDNA and PTPS deficiency-corrected iPSC cDNA. PCR was carried out with KOD-plus-Neo (Toyobo) and Veriti Thermal Cycler (Thermo Fisher Scientific).

### Western blot analysis

For cell collection, plates were washed with cold PBS, and cells were lysed on ice in RIPA buffer supplemented with protease inhibitor cocktail (Roche, Basel, Switzerland). The lysates were cleared by centrifugation at 12,000 g for 10 min at 4 °C. Protein concentration was determined by BCA assay (Pierce, Rockford, IL). About 20–30 µg of protein from the lysate were separated by 10–20% SDS-PAGE and then electrophoretically transferred to a PVDF membrane (Millipore, Darmastadt, Germany). Membranes were probed with the following primary antibodies: PTPS (GeneTex, San Antonio, TX) (110549, 1/1,000), DHPR (homemade polyclonal, 1/3,000), β-actin (Sigma-Aldrich, St. Louis, MO) (A5441, 1/5,000), TH (Millipore) (AB152, 1/1,000), βIII-tubulin (Cell Signaling Technology, Danvers, MA) (5568, 1/1,000). Membranes were then incubated with secondary peroxidase-conjugated antibody (GE Healthcare, Chicago, IL), and proteins were detected using ECL Prime Western Blotting Detection Reagent (GE Healthcare).

### Immunocytochemistry

Cells were fixed with 4% paraformaldehyde and blocked with PBS containing 5% foetal bovine serum. DAPI (4′, 6-diamidino-2-phenylindole) (Thermo Fisher Scientific) was used to label nuclei. Fluorescence imaging was performed using IN CELL Analyzer 6000 (GE Healthcare). The following primary antibodies were used: NANOG (ReproCELL, Yokohama, Japan) (RCAB0003P, 1/500), SSEA-4 (Millipore) (MAB4304, 1/1,000), βIII-tubulin (Millipore) (CBL412, 1/500), SOX-17 (R&D Systems, Minneapolis, MN) (AF1924, 1/300), αSMA (DAKO, Glostrup Denmark) (M0851, 1/100) and TH (Millipore) (AB152, 1/500).

### Dopamine release assay

iPSC-derived neuronal cells were washed twice with a low KCl (4.7 mM) solution. The medium was subsequently replaced with a high KCl solution (60 mM) for 20 min. The solution was collected, and concentration dopamine was determined by HPLC using a reverse-phase column and an electrochemical detector system (HTEC-500; Eicom, Kyoto, Japan).

### Measurement of pterins

iPSC-derived neuronal cells were lysed on ice in lysis buffer (0.4 M perchloric acid, 0.1 mM EDTA, 1 mM ascorbic acid). The cell lysate was deproteinized by centrifugation at 20,000 g for 10 min. Culture medium was deproteinized by the addition of 0.4 M perchloric acid, 1 mM EDTA, 10 mM ascorbic acid and following centrifugation. Pterin levels in the obtained supernatant were measured by HPLC with post-column oxidation and fluorescence detector system (34).

### Measurement of PTPS activity

PTPS activity was measured as previously described, with minor modifications (35). The cell lysate (20-40 μg protein) was incubated with 40 μM 7,8-dihydroneopterin triphosphate, 8 mM MgCl_2_ and 1 mM dithiothreitol for 1 h at 37ºC in the dark. The resultant 6-pyruvoyltetrahydropterin (PTP) was converted to pterin by iodine oxidation, and detected by the fluorometric HPLC method as described above.

### Statistical analysis

The data were analysed by Student’s *t* test, one-way ANOVA, followed by Dunnett’s multiple comparison test, or Tukey’s *post hoc* test using SAS 9.2.

## Supplementary Material

Supplementary Material is available at *HMG* online.

## Supplementary Material

Supplementary Data
