# Supplementary material for: Genetic and pharmacological correction of aberrant dopamine synthesis using patient iPSCs with BH4 metabolism disorders
Source: Hum Mol Genet. 2016 Oct 18;25(23):5188–97. doi: 10.1093/hmg/ddw339 (PMC5886044; doi:10.1093/hmg/ddw339)
Supplement: Supplementary Data [file ddw339_supp.docx]

**Figure S1.**


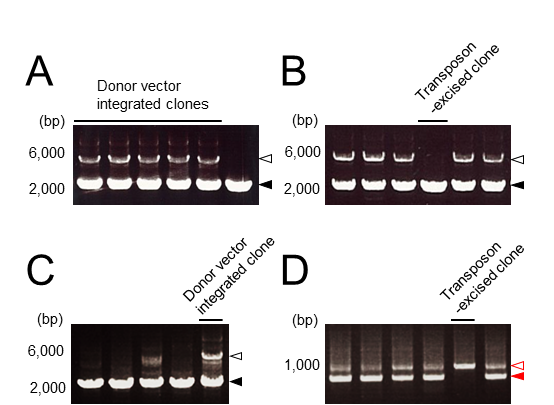


**(A, B)** PCR-based screening of PTPS deficiency-corrected iPSCs. Representative PCR results. Donor vector integrated clones were screened by PCR with primers 1, 2 (A) and transposon-excised clone was screened by PCR with primers 1, 2 (B). Black solid arrowheads indicate original or excised allele. Black open arrowheads indicate integrated allele.

**(C, D)** PCR-based screening of DHPR deficiency-corrected iPSCs. Representative PCR results. Donor vector integrated clone was screened by PCR with primers 1, 2 (C) and transposon-excised clone was screened by PCR with primers 3, 4, 5 (D). Black solid arrowhead indicates original allele. Black open arrowhead indicates integrated allele. Red solid arrowhead indicates integrated allele. Red open arrowhead indicates original or excised allele.

**Figure S2.**


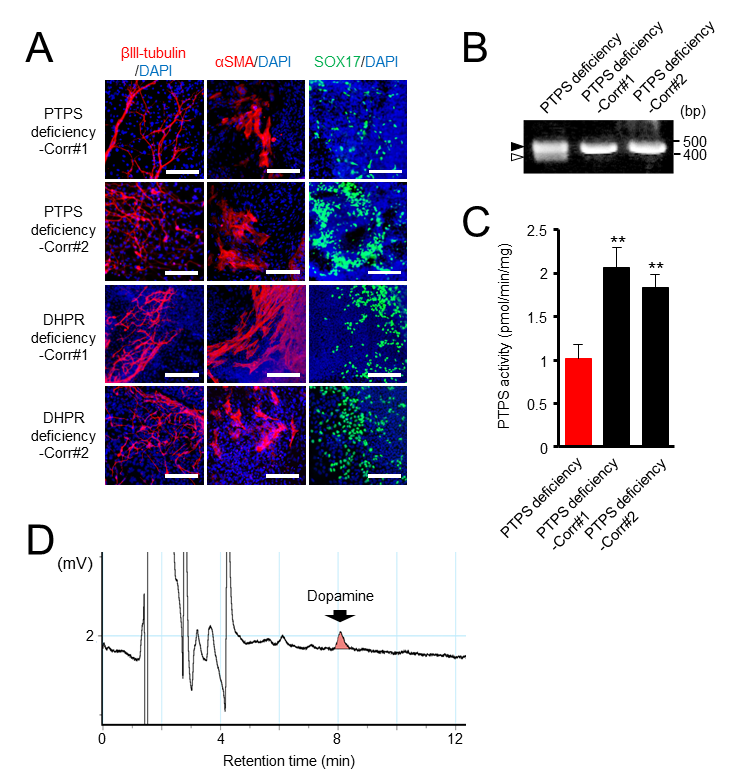


**(A)** In vitro differentiation of PTPS- and DHPR deficiency-corrected iPSCs to three germ layers: βIII-tubulin (ectoderm), αSMA (mesoderm), and SOX17 (endoderm). Scale bars: 100 μm.

**(B)** Migration on a 2.0% agarose gel of PTPS cDNAs of PTPS deficiency iPSCs and PTPS deficiency-corrected iPSCs. Black solid arrowhead indicates expected wild-type band. Black open arrowhead indicates splicing variant short band.

**(C)** PTPS activity in PTPS deficiency iPSCs and PTPS deficiency-corrected iPSCs. Data represent mean ± S.D. of n = 3. **p < 0.01, one-way ANOVA with Dunnett’s tests compared to PTPS deficiency iPSCs.

**(D)** Chromatogram of HPLC analysis of dopamine release by DA neuronal culture under high potassium stimulation.


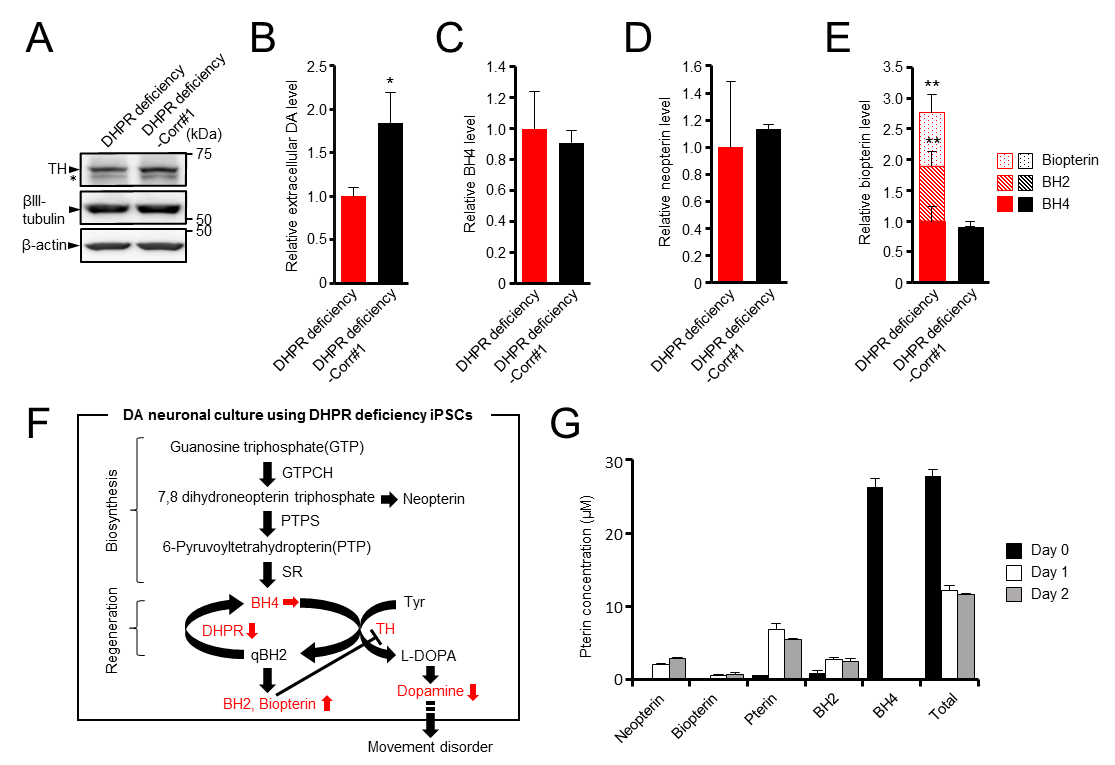


**Figure S3.**

**(A)** Western blot analysis of DA neuronal cultures using DHPR deficiency iPSCs and DHPR deficiency-corrected iPSCs for TH, βIII-tubulin and β-actin protein levels. Asterisk indicates non-specific band.

**(B)** Relative level of high potassium evoked-extracellular DA release in DA neuronal cultures using DHPR deficiency iPSCs and DHPR deficiency-corrected iPSCs. Quantification indicates mean ± S.D. of n = 3. *p < 0.05, Student’s *t* test.

**(C, D, E)** Pterin analysis in DA neuronal cultures using DHPR deficiency iPSCs and DHPR deficiency-corrected iPSCs: BH4 (C), Neopterin (D), Total biopterin (BH4 + BH2 + Biopterin) (E). Quantifications represent mean ± S.D. of n = 3. **p < 0.01, Student’s *t* test.

**(F)** Metabolic pathway of BH4 and DA synthesis in DHPR deficiency. c.176C>A variant in *QDPR* gene causes increase of BH2 amount. Accumulated BH2 leads to reduced DA level via inhibition of TH. Enzymes are indicated by the following abbreviations: GTPCH, GTP cyclohydrolase I; PTPS, 6-pyruvoyltetrahydropterin synthase; SR, sepiapterin reductase; DHPR, dihydropteridine reductase; TH, tyrosine hydroxylase

**(G)** Pterin contents in culture medium. Quantification indicates mean ± S.D. of n = 3.

**Figure S4.**


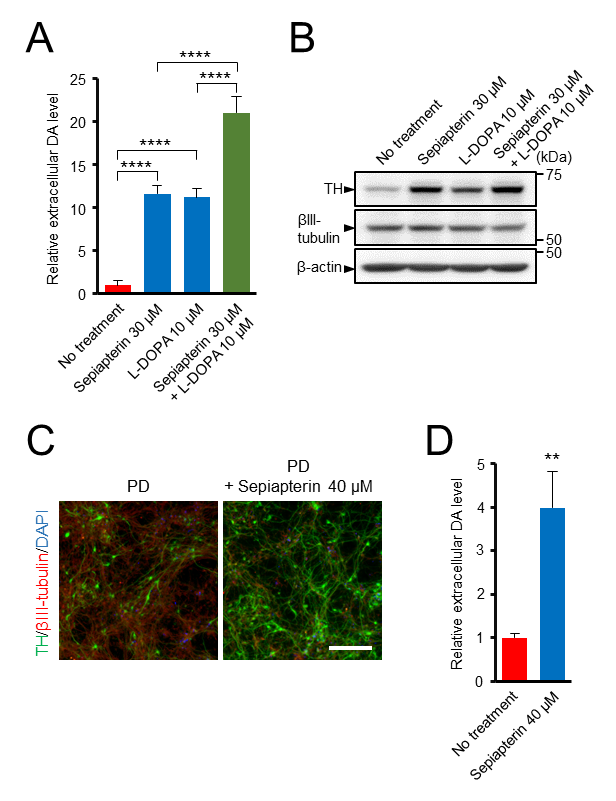


**(A, B)** Additive effect of 10 μM L-DOPA and 30 μM sepiapterin in DA neuronal culture using PTPS deficiency iPSCs. Relative level of high potassium evoked-extracellular DA release (A). Western blot analysis for TH, βIII-tubulin and β-actin protein levels (B). Quantification represents mean ± S.D. of n = 3. ****p < 0.0001, one-way ANOVA with Tukey’s post hoc test.

**(C, D)** Effect of sepiapterin in PD DA neuronal culture. PD DA neuronal cultures treated with DMSO or 40 μM sepiapterin were stained for TH, βIII-tubulin and DAPI (C). Scale bar: 100 μm. Relative level of high potassium-evoked extracellular DA release (D). Quantification represents mean ± S.D. of n = 3. **p < 0.01, Student’s *t* test.

**Table S1. Overview of generated patient-derived iPSCs.**

|  | **Gene** | **Variant** | **Sex** | **Age of biopsy** | **Reprogramming factors** |
| --- | --- | --- | --- | --- | --- |
| **PTPS deficiency** | *PTS* | c.243G>A/c.259C>T | female | 4 | OCT3/4, SOX2, KLF4, L-MYC, LIN28　and p53 carboxy-terminal dominant-negative fragment. |
| **DHPR deficiency** | *QDPR* | c.52G>T/c.176C>A | male | 15 | OCT3/4, SOX2, KLF4, L-MYC, LIN28　and p53 carboxy-terminal dominant-negative fragment. |
| **Parkinson's disease** | *GBA* | RecNci1/WT | female | 64 | OCT3/4, SOX2, KLF4, L-MYC, LIN28 and p53 carboxy-terminal dominant-negative fragment. |

**Table S2. Lists of primers used for quantitative RT-PCR.**

| **Gene** | **Forward** | **Reverse** |
| --- | --- | --- |
| *OCT3/4* | AGACCATCTGCCGCTTTGAG | GCAAGGGCCGCAGCTT |
| *NURR1* | CGAAACCGAAGAGCCCACAGGA | GGTCATAGCCGGGTTGGAGTCG |
| *LMX1A* | GATCCCTTCCGACAGGGTCTC | GGTTTCCCACTCTGGACTGC |
| *FOXA2* | TTCAGGCCCGGCTAACTCT | AGTCTCGACCCCCACTTGCT |
| *TH* | TCATCACCTGGTCACCAAGTT | GGTCGCCGTGCCTGTACT |
| *AADC* | CTCGGACCAAAGTGATCCAT | GTCTCTCTCCAGGGCTTCCT |
| *PTPS* | ACTGTTTGGGAAATGCAACA | TCATAACCATTCCCGTAGCA |
| *DHPR* | CCCGATGAACAGGAAATCA | CTGGATTAGGCTTCCTGAGC |
| *GAPDH* | GGTCGGAGTCAACGGATTTG | TCAGCCTTGACGGTGCCATG |
